# Lumbar disc degeneration is associated with modic change and high paraspinal fat content – a 3.0T magnetic resonance imaging study

**DOI:** 10.1186/s12891-016-1297-z

**Published:** 2016-10-21

**Authors:** Andrew J. Teichtahl, Donna M. Urquhart, Yuanyuan Wang, Anita E. Wluka, Richard O’Sullivan, Graeme Jones, Flavia M. Cicuttini

**Affiliations:** 1 Department of Epidemiology and Preventive Medicine, School of Public Health and Preventive Medicine, Monash University, Alfred Hospital, Melbourne, VIC 3004 Australia; 2 Baker IDI Heart and Diabetes Institute, Commercial Road, Melbourne, VIC 3004 Australia; 3 Healthcare Imaging Services, Epworth Hospital, Richmond, Melbourne, VIC 3121 Australia; 4 Department of Medicine, Central Clinical School, Monash University, Melbourne, VIC 3004 Australia; 5 Menzies Research Institute, Private bag 23, Hobart, TAS 7000 Australia

**Keywords:** Lumbar, Intervertebral disc, Disc degeneration, Modic, Muscle, Fat

## Abstract

**Background:**

Degenerative disc disease of the lumbar spine is common, with severe disease increasing the risk for chronic low back pain. This cross-sectional study examined whether disc degeneration is representative of a ‘whole-organ’ pathology, by examining its association with bone (vertebral endplate) and soft tissue (paraspinal muscle fat) abnormalities.

**Methods:**

Seventy-two community-based individuals unselected for low back pain, had Magnetic Resonance Imaging (MRI). Lumbosacral disc degeneration was determined via the Pfirrmann grading system, a validated method to assess the intervertebral disc, distinguishing the nucleus and annulus, the signal intensity and the height of the intervertebral disc. Modic change and high paraspinal muscle fat content was also measured from MRI.

**Results:**

Severe disc degeneration was associated, or tended to be associated with type 2 Modic change from L2 to L5 (OR range 3.5 to 25.3, *p* ≤ 0.06). Moreover, severe disc degeneration at all intervertebral levels was associated with or tended to be associated with high fat content of the paraspinal muscles (OR range 3.7 to 14.3, *p* ≤ 0.09).

**Conclusion:**

These data demonstrate that disc degeneration of the lumbar spine is commonly accompanied by Modic change and high fat content of paraspinal muscles, thus representing a ‘whole-organ’ pathology. Longitudinal studies are required to determine the temporal relationship between these structural abnormalities. Understanding this may have the potential to identify novel targets for the treatment and prevention of lumbosacral disc degeneration.

## Background

Degenerative disc disease is common in the lumbar spine, with one third of asymptomatic young women demonstrating abnormalities when assessed by Magnetic Resonance Imaging (MRI) [1]. In a case-control study of older adults with and without chronic low back pain, people with more severe disc degeneration had a two-fold increased risk of chronic low back pain than those without structural disc abnormalities [2]. Similarly, in a cross-sectional study, low back pain was found to be associated with several features of disc degeneration (dark nucleous pulposus and posterior and anterior bulge) [3].

Despite degenerative disc disease being considered to be a common finding, epidemiological studies have used varied measures to define disease. In histological studies these include granular changes, tear and cleft formation, chondrocyte proliferation, mucous degeneration and cell death [4], while macroscopic grading systems incorporate changes in the endplate and vertebral body, as well as nucleus and annulus [5]. Radiographic studies class disc degeneration by varying grades of joint space narrowing, endplate sclerosis and osteophytes [6, 7], while MRI studies have focussed on individual features including tears in the annulus [8], herniated nucleus [9] and height of the intervertebral disc [10]. Pfirrmann’s method (2001) [11] was endorsed as a valid and reliable method of assessing intervertebral disc degeneration using MRI in a systematic review of existing grading systems for lumbar disc degeneration [12]. The Pfirrmann system utilises a number of MRI features including the appearance of the disc structure, the signal intensity, intervertebral disc height and the distinction between the nucleus and the annulus, to give a 5 point grading system [11] (Table 1).

**Table 1:** Pfirrmann grading of lumbosacral disc degeneration ^a^

Grade	Structure	Distinction of nucleus and annulus	Signal intensity	Height of intervertebral disc
I	Homogenous, bright white	Clear	Hyperintense, isointense to cerebrospinal fluid	Normal
II	Inhomogenous with or without horizontal bands	Clear	Hyperintense, isointense to cerebrospinal fluid	Normal
III	Inhomogenous, grey	Unclear	Intermediate	Normal to slightly decreased
IV	Inhomogenous, grey to black	Lost	Intermediate to hypointense	Normal to moderately decreased
V	Inhomogenous, black	Lost	Hypointense	Collapsed disc space

^a^ Adapted from Pfirrmann et al. [11], whereby grades IV and V represent “severe intervertebral disc degeneration”

Although people with more severe disc degeneration have an increased risk for chronic low back pain [2, 3], whether disc degeneration is associated with other structural abnormalities is unclear. MRI has helped to better understand degenerative joint pathology. For instance, MRI studies of knee osteoarthritis have recognised that deleterious cartilage changes are accompanied by bone and soft tissue abnormalities, thus representing a ‘whole-organ’ disease [13]. Spinal degeneration has not been as well examined, and whether akin to the changes in hyaline cartilage in the knee, fibrocartilage degeneration of the intervertebral disc is associated with whole-organ spinal pathology is unclear. Vertebral endplate lesions (Modic change) may be analogous to subchondral bone sclerosis seen in osteoarthritis and may be an important association with disc degeneration [14–21]. Moreover, changes in the architecture of spinal musculature, in particular fat content [9, 22–26], may also be a key structural feature of spinal degeneration.

The aim of this cross-sectional MRI study was to determine the associations between intervertebral disc degeneration of the lumbar spine and Modic and paraspinal muscle fat content.

## Methods

### Participants

Seventy-two community-based individuals were recruited through local media and weight loss clinics as part of a study of obesity and musculoskeletal health. Participants were recruited without reference to whether they had or did not have low back pain. Participants were excluded if they had a history of malignancy, significant systemic condition (e.g. cerebrovascular accident, movement disorder, or connective tissue disease), or inability to understand English. A further exclusion criterion was any contraindication to MRI. Participants gave written informed consent. The study was approved by the Human Research Ethics Committees of the Alfred Hospital and Monash University.

### Magnetic resonance imaging

MRI (2011–2012) was performed using a 3.0-T magnetic resonance unit (MAGNETOM Verio, A Tim System; Siemens, Erlangen, Germany). The participants’ were positioned in supine and the following scans were performed: (1) sagittal T1 Turbo Spin Echo (TSE) imaging from T12 to the sacrum (time to recovery 670 ms; time to echo: 12 ms, slice thickness: 4 mm), (2) sagittal T2 TSE imaging from T12 to sacrum (time to recovery: 3000–3600 ms; time to echo: 87–114 ms, slice thickness: 4 mm), and (3) axial T2 TSE imaging from L1 to S1 (time to recovery: 3000–3600 ms; time to echo: 87–114 ms, slice thickness: 4 mm). Different readers independently measured each structural lesion (e.g. modic change, intervertebral disc and paraspinal muscle fat content), blinded to the results of other readers.

#### Degenerative intervertebral disc assessment

Intervertebral disc degeneration was assessed from T2-weighted sagittal images based on the Pfirrmann method [11] (Table 1). Grades 4 and 5 formed a “severe disc degeneration” group. The measurement was performed by one assessor who was trained to measure disc degeneration by a radiologist experienced in musculoskeletal MRI, blinded to the characteristics of the participants. Images were reassessed 1 week apart. The intra-rater reliability of the disc degeneration measures at each vertebral level was high, with intra-class correlation coefficients (ICCs) ranging from 0.88 to 0.94 for the I-V grading system.

#### Modic change

Modic change was classified according to the original system [21, 27] into 3 types:

- Type 1: hypointense on T1 and hyperintense on T2 images
- Type 2: hyperintense on T1 and isointense/hyperintense on T2 images
- Type 3: hypointense on both T1 and T2 images

Images were assessed in the sagittal plane. A predefined cut-off point for size was not used. The presence of modic change was defined based on signal changes on either side of the disc. Sixty randomly selected images were reassessed for Modic change by the same observer 1 week later. The ICC was found to be 0.74.

#### Paraspinal muscle fat content

Hyperintense regions of the paraspinal muscles observed on T2 axial images at the level of each lumbar intervertebral disc (L1/2, L2/3, L3/4 and L4/5) were considered to represent fat replacement [9] and categorised based on a previously validated grading method; grade 0: no fat, grade 1: 1–10 % fat, grade 2: 11–50 % fat and grade 3: >50 % fat [9]. High fat content was defined as greater than 50 % of the muscle. The intra-observer ICC for the paraspinal muscle fat content for both multifidus and erector spinae was 0.99. To provide a measure of high paraspinal muscle fat content in the lumbar spine, a dichotomous outcome was created. This measure scored participants demonstrating >50 % of a muscle in at least four spinal levels on either the right and/or left sides (with the total possible score being 8).

### Anthropometric data

Height was measured to the nearest 0.1 cm using a stadiometer. Weight was measured to the nearest 0.1 kg using a single pair of electronic scales. BMI (kg m^−2^) was calculated.

### Chronic pain and disability

The Chronic Pain Grade Questionnaire was administered at the time of MRI in 2011–2012 to obtain information on low back pain intensity over the past 6 months. The Chronic Pain Grade Questionnaire is a reliable and valid instrument for use in population surveys of low back pain [28, 29]. The questionnaire includes seven questions from which a pain intensity and disability subscale score are calculated. Subscale scores for pain intensity and disability are combined to calculate a chronic pain grade that enables classification of chronic pain into 5 hierarchical categories: grades 0 (no pain) to 4 (high disability, severely limiting) as previously described [28, 29]. High intensity pain/disability was defined as being of either grade 2 (low disability but high intensity), grade 3 (high disability, moderately limiting) or grade 4 (high disability, severely limiting).

### Statistical analyses

Binary logistic regression was used to examine the relationships between all exposures (Modic change and fat content of paraspinal muscles) and the prevalence of severe intervertebral disc degeneration, adjusted for age, gender, BMI and high intensity pain and or disability. A *p*-value of less than 0.05 (two-tailed) was regarded as statistically significant. All analyses were performed using the SPSS statistical package (standard version 20.0 SPSS, Chicago, IL, USA). With 72 subjects, our study had 80 % power to detect an odds ratio as low as 2.8, assuming the prevalence of severe intervertebral disc degeneration being 20 %, α = 0.05, and 2-sided significance.

## Results

The characteristics of the 72 participants are shown in Table 2. The mean (± standard deviation) age of the cohort was 48.7 ± 8.3 years, comprising 49 (68.1 %) females. The prevalence of severe intervertebral disc degeneration was low at L1/2 (4.2 %) but increased at more inferior lumbosacral levels (L2/3 11.1 %, L3/4 15.3 %, L4/5 31.9 %, L5/S1 30.6 %). Similarly, the prevalence of Modic type 2 change was low at the L1/2 vertebral level (5.6 %) but increased at more inferior lumbosacral levels (L2/3 11.1 %, L3/4 22.2 %, L4/5 29.2 %, L5/S1 23.6 %). No participants demonstrated a Modic type 3 change. The prevalence of Modic type 1 change was low (*n* = 4), and was therefore not the focus of subsequent analyses. Sixteen participants (22.5 %) had >50 % fat content of either or both multifidus and erector spinae at 4 or more lumbar levels (L1/2, L2/3, L3/4 or L4/5).

**Table 2:** Subject demographics (*n* = 72) ^a^

Age (years)	48.7 (8.3)	
Gender (n, % female)	49 (68.1)	
BMI (kgm^−2^)	29.2 (7.9)	
Chronic low back pain grade, n (%)		
*0 – Pain free*	14 (19.2)	
*1 – Low disability, low intensity*	44 (60.3)	
*2 – Low disability, high intensity*	5 (6.8)	
*3 – High disability, moderately limiting*	5 (6.8)	
*4 – High disability, severely limiting*	5 (6.8)	
High intensity pain and or disability, n (%)	15 (20.5)	
Severe intervertebral disc degeneration n (%)		
*L1/2*	3 (4.2)	
*L2/3*	8 (11.1)	
*L3/4*	11 (15.3)	
*L4/5*	23 (31.9)	
*L5/S1*	22 (30.6)	
Modic (type 2) change in vertebrae adjacent to disc n (%)		
*L1/L2*	4 (5.6)	
*L2/L3*	8 (11.1)	
*L3/L4*	16 (22.2)	
*L4/L5*	21 (29.2)	
*L5/S1*	17 (23.6)	
Fat content (>50 %) at number of lumbar spinal levels, n (%)		
	Multifidus	Erector spinae
0	42 (57.7)	35 (47.9)
1	8 (11.3)	23 (32.4)
2	12 (16.9)	3 (4.2)
3	4 (5.6)	5 (7.0)
4	6 (8.5)	6 (8.5)
High paraspinal muscle fat content, n (%)^b^	16 (22.5)	

^a^Results presented as mean (standard deviation) unless otherwise stated

^b^Derived from participants as having >50 % of a muscle (multifidus or erector spinae) replaced by fat in ≥4 lumbar spinal levels, with the total possible score being 8

The associations between severe intervertebral disc degeneration and Modic change in the adjacent vertebrae are shown in Table 3. In univariate analyses, Modic change in the L2/3 and L3/4 vertebrae was significantly associated with accompanying severe intervertebral disc degeneration (all *p* ≤ 0.05) and approached statistical significance at the L4/5 level (*p* = 0.07). After adjusting for age, gender, BMI and high pain and or disability, these relationships were significant or approached statistical significance at L2/3 (OR 25.3, 95 % CI 1.4 to 471.9, *p* = 0.03), L3/4 (OR 4.5, 95 % CI 1.0 to 21.0, *p* = 0.06) and L4/5 (OR 3.5, 95 % CI 1.0 to 12.5, *p* = 0.05). There was no association between Modic change at the L5/S1 level and severe disc degeneration at that corresponding level (OR 1.2, 95 % CI 0.3, 3.9, *p* = 0.82).

**Table 3:** The prevalence of severe intervertebral disc degeneration in relation to Modic change in the adjacent vertebrae

Disc level	Prevalence of severe intervertebral disc degeneration			
	Univariate OR (95 % CI)	P	Multivariate^a^ OR (95 % CI)	P
L2/3	7.1 (1.3, 38.7)	0.02	25.3 (1.4, 471.9)	0.03
L3/4	3.8 (1.0, 14.7)	0.05	4.5 (1.0, 21.0)	0.05
L4/5	2.7 (0.9, 7.7)	0.07	3.5 (1.0, 12.5)	0.05
L5/S1	1.3 (0.4, 4.2)	0.63	1.2 (0.3, 3.9)	0.82

^a^ Adjusted for age, gender, BMI and high intensity pain and or disability

The associations between high fat content of paraspinal muscles and severe intervertebral disc degeneration are shown in Table 4. High fat content of the paraspinal muscles were associated with severe degenerative disease at L2/3 (OR 9.9, 95 % CI 1.2 to 81.5, *p* = 0.03), L3/4 (OR 14.3, 95 % CI 2.2 to 91.2, *p* = 0.005), L5/S1 (OR 4.5, 95 % CI 1.1 to 18.8, *p* = 0.04) with results approaching statistical significance at L4/5 (OR 3.7, 95 % CI 0.8 to 16.0, *p* = 0.09) after adjusting for age, gender, BMI and high pain and or disability.

**Table 4:** The prevalence of severe intervertebral disc degeneration in relation to high fat content in paraspinal muscles

	Univariate OR (95 % CI)	P	Multivariate OR (95 % CI)	P
L2/3	15.9 (2.8, 90.3)	0.002	9.9 (1.2, 81.5)	0.03
L3/4	17.3 (3.8, 79.4)	<0.001	14.3 (2.2, 91.2)	0.005
L4/5	5.4 (1.6, 17.7)	0.005	3.7 (0.8, 16.0)	0.09
L5/S1	6.0 (1.8, 19.8)	0.003	4.5 (1.1, 18.8)	0.04

Adjusted for age, gender, BMI, and high intensity pain and or disability

## Discussion

This cross-sectional study has demonstrated that severe disc degeneration of the lumbar spine is accompanied by other structural lesions. In particular, the presence of severe disc degeneration was associated with adjacent Modic type 2 change and high fat content of the paraspinal muscles. These data highlight that akin to degenerative processes such as knee osteoarthritis, spinal degeneration appears to be a ‘whole-organ’ disease, affecting cartilage (intervertebral disc), bone (Modic change) and soft tissues (paraspinal musculature).

As in a previous study [30], we found that the greatest burden of degenerative disc disease was in the low lumbar (L4/5) and lumbosacral (L5/S1) spine. Degenerative disc disease was uncommon at L1/2, with numbers too small to meaningfully analyse (4.2 %). Since disc degeneration and Modic changes are more common in the low lumbar spine, a previous study focused on the L5/S1 region in 228 male workers [31]. Consistent with the previous study, we did not observe a significant association between Modic change and the risk of severe L5/S1 degenerative disc disease. Since L5/S1 is a transitional point in the spine, it may be that the association between Modic change and degenerative disc disease at this level differs from the rest of the lumbar spine. Indeed, we have demonstrated that Modic type 2 change was associated with severe disc degeneration at most other lumbar levels. In another previous study of 108 surgical patients with lumbar degenerative disc disease graded by the Pfirrmann system, Modic changes correlated with the grade of disc degeneration [32]. However in this previous study, it was unclear whether Modic change was accompanied by degenerative disc disease at the *same* vertebral level. To our knowledge, the current study is the first study to present such data. Although this may infer a local interaction between the two structural abnormalities, the mechanism accounting for the co-existence of degenerative disc disease and accompanying Modic type 2 change is unclear. It may be that disc degeneration reduces the shock absorbing capability of the vertebrae, resulting in Modic change. Alternatively, it may be that disruption to the vertebral endplates, such as Modic changes, impedes nutritional support of the intervertebral disc, causing subsequent disc degeneration. In a previous study, endplate cartilage damage increased with age and produced considerable changes in diffusion [33]. Longitudinal studies are required to determine which structural features may be the antecedent event in the natural history of degenerative disc disease.

In the current study, we have demonstrated that a high fat content of paraspinal muscles is associated with severe intervertebral disc degeneration at each intervertebral level. Muscle atrophy is, in part, characterised by fat infiltration [22, 24]. Histological studies have demonstrated concordance between intermuscular adipose tissue detected by MRI and intra-operative specimens of paraspinal muscles [34], with other studies corroborating MRI as a valid method of identifying the amount of fat in skeletal muscle [35, 36]. Nevertheless, to our knowledge, only one MRI study has examined the association between paraspinal muscle fat infiltration and intervertebral disc degeneration. A retrospective study of 78 participants showed only a tendency toward multifidus muscle atrophy (defined by the degree of fat and fibrous tissue replacement) being associated with nerve root compression, herniated nucleus pulposus and the number of degenerated discs [9]. The mechanism accounting for the relationship between a high fat content of paraspinal muscles and severe degenerative disc disease is speculative. It is possible that degenerative disc disease causes pain and reduced activity levels, resulting in fat replacement of paraspinal muscles. We have however adjusted our results for people with high intensity pain and or disability, suggesting that the observed associations are independent of the potential confounding effect of varied physical activity levels. Equally plausible is the potential for fatty replacement of paraspinal muscles to reduce segmental stability of the spine, causing disc degeneration. Longitudinal studies will help to address such issues.

A limitation of this study was its cross-sectional design, and thus it cannot be determined whether associations between degenerative disc disease and other structural features are a cause or result of one another. We used the Pfirrmann grading system to assess disc degeneration. While this 5 grade system has difficulty discriminating disc pathology in the elderly spine (mean age 73 years; range 67 to 83 years) and requires a modified grading system [37], our cohort was relatively young (mean age 48.7 (±8.3) years). Additionally, we used a semi-quantitative method of assessing fat replacement of paraspinal muscle based on previously employed methods [9, 22, 38] and further adapted this system whereby a high fat content within the paraspinal compartment necessitated a participant demonstrating >50 % of a muscle in at least four spinal levels in any combination of the right and left sides (with the total possible score being 8). We combined the erector spinae and multifidus muscles into the posterior compartment in an attempt to identify individuals with poor quality supporting musculature, rather than a focus on individual muscles. This is a conservative approach and any potential misclassification of high paraspinal fat content is likely to have reduced our ability to demonstrate statistically significant associations. Similarly, any potential misclassification of disc degeneration in this study, whereby grades 4 and 5 represented severe disease, is likely to be non-differential and have underestimated any of the associations observed in this study. Moreover, although we have asserted that our MRI measure captured replacement of muscle with fat based on the concordance between intermuscular adipose tissue detected by MRI and intra-operative specimens of paraspinal muscles [34], it is possible that other fibrous non-muscular elements may have been captured by this assessment. Furthermore, this study recruited community-based subjects. The presence of chronic pain was not required for inclusion in this study, which likely accounts for the predominance of type 2, rather than type 1 Modic change. Moreover, inclusion criteria necessitating the need for chronic pain can be problematic, since avoidance behaviours and psychosocial variability are likely to be apparent in chronic diseases [39, 40]. For instance, it has been documented that fear of movement is a common occurrence among people with chronic low back pain [41], and selection of such subgroups in study designs may lead to selection bias. Instead, we chose to measure community-based subjects with the aim of capturing a spectrum of spinal abnormalities. Nevertheless, we have adjusted our analyses for the presence of high pain and or disability.

## Conclusion

This study has demonstrated that severe disc degeneration of the lumbar spine is accompanied by other structural lesions. In particular, the presence of severe disc degeneration is associated with adjacent Modic type 2 change and high fat content of the paraspinal muscles. Although longitudinal studies are required to determine the temporal relationship between these changes, these data highlight the ‘whole-organ’ disease occurring in disc degeneration. Understanding this will have the potential to identify novel targets for the treatment and prevention of lumbosacral disc degeneration.

## Abbreviations

BMI: Body mass index
CI: Confidence interval
ICC: Intra-class correlation coefficient
IL: Interleukin
MRI: Magnetic resonance imaging
OR: Odds ratio
TNF: Tumour necrosis factor

## References

[1] Powell MC, Wilson M, Szypryt P, Symonds EM, Worthington BS (1986). Prevalence of lumbar disc degeneration observed by magnetic resonance in symptomless women. Lancet.

[2] Hicks GE, Morone N, Weiner DK (2009). Degenerative lumbar disc and facet disease in older adults: prevalence and clinical correlates. Spine (Phila Pa 1976).

[3] Luoma K, Riihimaki H, Luukkonen R, Raininko R, Viikari-Juntura E, Lamminen A (2000). Low back pain in relation to lumbar disc degeneration. Spine.

[4] Boos N, Weissbach S, Rohrbach H, Weiler C, Spratt KF, Nerlich AG (2002). Classification of age-related changes in lumbar intervertebral discs: 2002 Volvo Award in basic science. Spine (Phila Pa 1976).

[5] Thompson JP, Pearce RH, Schechter MT, Adams ME, Tsang IK, Bishop PB (1990). Preliminary evaluation of a scheme for grading the gross morphology of the human intervertebral disc. Spine (Phila Pa 1976).

[6] Mimura M, Panjabi MM, Oxland TR, Crisco JJ, Yamamoto I, Vasavada A (1994). Disc degeneration affects the multidirectional flexibility of the lumbar spine. Spine (Phila Pa 1976).

[7] Lane NE, Nevitt MC, Genant HK, Hochberg MC (1993). Reliability of new indices of radiographic osteoarthritis of the hand and hip and lumbar disc degeneration. J Rheumatol.

[8] Yu S, Haughton VM, Sether LA, Ho KC, Wagner M (1989). Criteria for classifying normal and degenerated lumbar intervertebral disks. Radiology.

[9] Kader DF, Wardlaw D, Smith FW (2000). Correlation between the MRI changes in the lumbar multifidus muscles and leg pain. Clin Radiol.

[10] Urquhart DM, Kurniadi I, Triangto K, Wang Y, Wluka AE, O’Sullivan R, Jones G, Cicuttini FM (2014). Obesity is Associated With Reduced Disc Height in the Lumbar Spine but not at the Lumbosacral Junction. Spine.

[11] Pfirrmann CW, Metzdorf A, Zanetti M, Hodler J, Boos N (2001). Magnetic resonance classification of lumbar intervertebral disc degeneration. Spine (Phila Pa 1976).

[12] Kettler A, Wilke HJ (2006). Review of existing grading systems for cervical or lumbar disc and facet joint degeneration. Eur Spine J.

[13] Teichtahl AJ, Wluka AE, Davies-Tuck ML, Cicuttini FM (2008). Imaging of knee osteoarthritis. Best Pract Res Clin Rheumatol.

[14] Toyone T, Takahashi K, Kitahara H, Yamagata M, Murakami M, Moriya H (1994). Vertebral bone-marrow changes in degenerative lumbar disc disease. An MRI study of 74 patients with low back pain. J Bone Joint Surg (Br).

[15] Mitra D, Cassar-Pullicino VN, McCall IW (2004). Longitudinal study of vertebral type-1 end-plate changes on MR of the lumbar spine. Eur Radiol.

[16] Kuisma M, Karppinen J, Niinimaki J, Ojala R, Haapea M, Heliovaara M, Korpelainen R, Taimela S, Natri A, Tervonen O (2007). Modic changes in endplates of lumbar vertebral bodies: prevalence and association with low back and sciatic pain among middle-aged male workers. Spine (Phila Pa 1976).

[17] Albert HB, Manniche C (2007). Modic changes following lumbar disc herniation. Eur Spine J.

[18] Modic MT (2007). Modic type 1 and type 2 changes. J Neurosurg Spine.

[19] Weishaupt D, Zanetti M, Hodler J, Boos N (1998). MR imaging of the lumbar spine: prevalence of intervertebral disk extrusion and sequestration, nerve root compression, end plate abnormalities, and osteoarthritis of the facet joints in asymptomatic volunteers. Radiology.

[20] Kjaer P, Korsholm L, Bendix T, Sorensen JS, Leboeuf-Yde C (2006). Modic changes and their associations with clinical findings. Eur Spine J.

[21] Modic MT, Masaryk TJ, Ross JS, Carter JR (1988). Imaging of degenerative disk disease. Radiology.

[22] Kjaer P, Bendix T, Sorensen JS, Korsholm L, Leboeuf-Yde C (2007). Are MRI-defined fat infiltrations in the multifidus muscles associated with low back pain?. BMC Med.

[23] Fischer MA, Nanz D, Shimakawa A, Schirmer T, Guggenberger R, Chhabra A, Carrino JA, Andreisek G (2013). Quantification of muscle fat in patients with low back pain: comparison of multi-echo MR imaging with single-voxel MR spectroscopy. Radiology.

[24] Mengiardi B, Schmid MR, Boos N, Pfirrmann CW, Brunner F, Elfering A, Hodler J (2006). Fat content of lumbar paraspinal muscles in patients with chronic low back pain and in asymptomatic volunteers: quantification with MR spectroscopy. Radiology.

[25] Teichtahl AJ, Urquhart DM, Wang Y, Wluka AE, O’Sullivan R, Jones G, Cicuttini FM (2015). Physical inactivity is associated with narrower lumbar intervertebral discs, high fat content of paraspinal muscles and low back pain and disability. Arthritis Res Ther.

[26] Teichtahl AJ, Urquhart DM, Wang Y, Wluka AE, Wijethilake P, O’Sullivan R, Cicuttini FM (2015). Fat infiltration of paraspinal muscles is associated with low back pain, disability, and structural abnormalities in community-based adults. Spine J.

[27] Modic MT, Steinberg PM, Ross JS, Masaryk TJ, Carter JR (1988). Degenerative disk disease: assessment of changes in vertebral body marrow with MR imaging. Radiology.

[28] Von Korff M, Ormel J, Keefe FJ, Dworkin SF (1992). Grading the severity of chronic pain. Pain.

[29] Smith BH, Penny KI, Purves AM, Munro C, Wilson B, Grimshaw J, Chambers WA, Smith WC (1997). The Chronic Pain Grade questionnaire: validation and reliability in postal research. Pain.

[30] West W, West KP, Younger EN, Cornwall D (2010). Degenerative disc disease of the lumbar spine on MRI. West Indian Med J.

[31] Kuisma M, Karppinen J, Haapea M, Niinimaki J, Ojala R, Heliovaara M, Korpelainen R, Kaikkonen K, Taimela S, Natri A, Tervonen O (2008). Are the determinants of vertebral endplate changes and severe disc degeneration in the lumbar spine the same? A magnetic resonance imaging study in middle-aged male workers. BMC Musculoskelet Disord.

[32] Yu LP, Qian WW, Yin GY, Ren YX, Hu ZY (2012). MRI assessment of lumbar intervertebral disc degeneration with lumbar degenerative disease using the Pfirrmann grading systems. PLoS One.

[33] Rajasekaran S, Babu JN, Arun R, Armstrong BR, Shetty AP, Murugan S (2004). ISSLS prize winner: A study of diffusion in human lumbar discs: a serial magnetic resonance imaging study documenting the influence of the endplate on diffusion in normal and degenerate discs. Spine (Phila Pa 1976).

[34] Rossi A, Zoico E, Goodpaster BH, Sepe A, Di Francesco V, Fantin F, Pizzini F, Corzato F, Vitali A, Micciolo R, Harris TB, Cinti S, Zamboni M (2010). Quantification of intermuscular adipose tissue in the erector spinae muscle by MRI: agreement with histological evaluation. Obesity (Silver Spring).

[35] Mitsiopoulos N, Baumgartner RN, Heymsfield SB, Lyons W, Gallagher D, Ross R (1998). Cadaver validation of skeletal muscle measurement by magnetic resonance imaging and computerized tomography. J Appl Physiol (1985).

[36] Phoenix J, Betal D, Roberts N, Helliwell TR, Edwards RH (1996). Objective quantification of muscle and fat in human dystrophic muscle by magnetic resonance image analysis. Muscle Nerve.

[37] Griffith JF, Wang YX, Antonio GE, Choi KC, Yu A, Ahuja AT, Leung PC (2007). Modified Pfirrmann grading system for lumbar intervertebral disc degeneration. Spine (Phila Pa 1976).

[38] Parkkola R, Rytokoski U, Kormano M (1993). Magnetic resonance imaging of the discs and trunk muscles in patients with chronic low back pain and healthy control subjects. Spine (Phila Pa 1976).

[39] Philips HC (1987). Avoidance behaviour and its role in sustaining chronic pain. Behav Res Ther.

[40] Gatchel RJ, Polatin PB, Mayer TG (1995). The dominant role of psychosocial risk factors in the development of chronic low back pain disability. Spine (Phila Pa 1976).

[41] Vlaeyen JW, Kole-Snijders AM, Boeren RG, van Eek H (1995). Fear of movement/(re)injury in chronic low back pain and its relation to behavioral performance. Pain.

